# Insect Rearing Techniques for Biological Control Programs, a Component of Sustainable Agriculture in Brazil

**DOI:** 10.3390/insects13010105

**Published:** 2022-01-17

**Authors:** José Roberto Postali Parra, Aloisio Coelho

**Affiliations:** Departamento de Entomologia e Acarologia, Escola Superior de Agricultura Luiz de Queiroz (ESALQ), Universidade de São Paulo (USP), Av. Pádua Dias, 11, Piracicaba 13418-900, SP, Brazil; jrpparra@usp.br

**Keywords:** sustainability, agriculture 4.0, eco-friendly technology

## Abstract

**Simple Summary:**

This review describes the advances in BC for use in open fields in Brazil. These advances make our country a model for this type of pest control, especially since 1980, with the development of improved rearing techniques. In association with private companies, the use of BC has grown more than in the rest of the world, advancing by about 10–15% each year.

**Abstract:**

This article describes the importance of rearing insects, whether on a small scale for research or a large scale for mass rearing, for use in biological control (BC) programs with macro-organisms. These inter- or multidisciplinary research programs are necessarily long-term and depend on rearing techniques for their complete development. Some successful examples of BC in Brazil are presented, including case studies of *Trichogramma* spp. These required broad bioecological studies that provided the basis for both mass rearing and transfer of the necessary technology to farmers. This has allowed Brazil to occupy a leadership position in biological control in “Open Fields”. For example, about three million ha are being treated with *Trichogramma galloi* (a native parasitoid), and about three and a half million ha with *Cotesia flavipes* (an exotic parasitoid) to control *Diatraea saccharalis*, the sugarcane borer. These natural enemies are produced by commercial firms, or by laboratories in sugar and alcohol plants themselves, in the case of *C. flavipes*.

## 1. Introduction

Brazil is a leader in tropical agriculture, with a forecasted production of 289.7 million tons of agricultural commodities in 2020–2021, reached through technology developed in this country [1]. The mindset of using agrochemicals for pest control prevailed among Brazilian farmers for a long period, although Brazil imported the first natural enemy in 1921 for use in a classical biological control (BC) program [2,3]. This first program used the species *Encarsia berlesei* Howard (Hym.: Aphelinidae, then assigned to the genus Prospaltella), imported from the USA, to control *Pseudaulacaspis pentagona* (Targioni) (Hem.: Diaspididae) on peaches. No control resulted, due to the lack of techniques for rearing the pest and the imported natural enemy, a failure that continued until 1940, for other imported species, for the same reason [2]. Biological control in Brazil advanced with the establishment of graduate programs in the 1960s, coincident with the establishment of the Entomological Society of Brazil in 1972. BC advanced from 1980 to 2010 onward, with extension courses on insect rearing techniques for BC, conducted in different states by the team from the Department of Entomology and Acarology of the University of São Paulo (USP), Luiz de Queiroz College of Agriculture (ESALQ).

In 1976, integrated pest management (IPM) was established by Smith et al. (1976) [4]. These programs require the production of insects for different areas, i.e., for the development of measures to maintain pest numbers below the level of economic damage. Mastery of techniques for insect rearing has led to significant advances in several areas, including bioecology, insecticide selectivity, insect pheromones, pest and natural enemy zoning, resistance management, transgenic plants, molecular studies, insect pathology (micro-organisms), and symbionts, but mainly mass rearing of insects for BC with macroorganisms.

Along with the millions of hectares (ha) treated with micro-organisms in Brazil, certain crops, such as sugarcane, are now being treated with macroorganisms, such as *Trichogramma galloi* Zucchi (Hym.: Trichogrammatidae) (a native parasitoid) and *Cotesia flavipes* (Cameron) (Hym.: Braconidae) (an exotic parasitoid), on 3 and 3.5 million ha, respectively. Almost all the parasitoids (90–95%) are released by drones at a cost competitive with chemicals. Nowadays, species of *Trichogramma* Westwood (Hym.: Trichogrammatidae) are used in varying numbers of 50 to 400 thousand individuals per ha in cotton, corn, avocado, citrus, soybean, tomato, and other crops to control lepidopterans [3]. Use of these tiny wasps is made possible by the production of eggs of their factitious host *Anagasta kuehniella* Zeller (Lep.: Pyralidae), in quantities of up to 30–40 kg of eggs per day (1 g = 36,000 eggs).

Advances in artificial diets, including multidimensional and nutrigenomic studies, would substantially improve rearing techniques for different groups of Lepidoptera, Coleoptera, and Diptera, and for other orders. An example of advance on the last year, nevertheless without the mentioned technics, is the lyophilized green bean-based diet for the main soybean pest, the brown stink bug *Euschistus heros* (F.) (Hem.: Pentatomidae), to produce its egg parasitoid, the natural enemy *Telenomus podisi* Ashmead (Hym.: Scelionidae) [5]. Commercial-scale programs to control citrus pests, using, for example, the egg-parasitoid wasp *Ageniaspis citricola* Logvinoskaya (Hym.: Encyrtidae) to control the leafminer *Phyllocnistis citrella* Stainton (Lep.: Gracillariidae) and the ectoparasitoid wasp *Tamarixia radiata* (Waterston) (Hym.: Eulophidae) to control the psyllid *Diaphorina citri* Kuwayama (Hem.: Psyllidae), are, or have been, routinely used in citrus production, the latter produced on orange jessamine [*Murraya paniculata* (L.) (Sap.: Rutacaeae)].

This review describes the advances in BC, for use in open fields in Brazil. These advances have made our country a model for this type of pest control, especially since 1980, with the development of improved rearing techniques. In association with private companies, the use of BC has grown more than in the rest of the world, advancing by about 10–15% each year. BC in Brazil includes the use of both micro- and macro-organisms, the latter being the subject of this review [6,7].

## 2. Biological Control and Insect Rearing

Based on current knowledge of the development of biological control programs, it is necessary to rear the target pest and its natural enemy, as the “in vitro” rearing studies started in the 1980s, with the presentation in China (Guangzhou) [8] of a diet for *Trichogramma dendrolimi* Matsumura (Hym.: Trichogrammatidae), did not evolve as expected (Cônsoli and Grenier, 2010) [9]. Although Brazil produced species of *Trichogramma* (*T. galloi*, *Trichogramma pretiosum* Riley, *Trichogramma atopovirilia* Oatman & Platner) and *Habrobracon hebetor* (Say) (Hym.: Braconidae), viable rearing “in vitro” on a mass scale was not achieved. Despite advances in the field of nutrigenomics and multidimensional systems for artificial diets [10,11,12], it is still necessary to rear two species of insects, the pest, and its natural enemy.

Considering the procedures for biological control, that is, introduction (classical biological control), conservation (natural or conservative biological control), multiplication (augmentative or applied biological control), and external management (for *D. citri*, transmitter of HLB in citrus [13]), in every case, it is necessary to rear the natural enemy in the laboratory. Natural enemies are needed in varying numbers, and different life stages may be required for particular BC programs.

In the case of classical biological control, during the initiation of BC as a control method, for lack of rearing techniques, the releases were conducted with small numbers of insects (inoculative releases), which required some time for the population of the natural enemy to increase and consequently served only for perennial or semi-perennial crops. The classic case is *Rodolia cardinalis* (Mulsant) (Col.: Coccinellidae) (currently the species is referred as *Novius cardinalis*; however, given the consecration of the fact, the species name *Rodolia cardinalis* is considered in the present work), used in 1888 to control *Icerya purchasi* Maskell (Hem.: Monophlebidae), which was brought from Australia, the place of origin of the mealybug that had become a pest of citrus in California. This is the first case of true success of modern BC in the world.

In the case of conservative biological control, in which one attempts to manage the habitat, in order to conserve existing control agents and, if possible, to increase them, the techniques used do not always need large numbers of insects for release. Selective products are used, also taking into account the resistance of pests to applied chemical products (resistance management) and provision of food (pollen) to the adults, for example.

In the case of external management, the foci of contamination of the bacteria that cause “Huanglongbing” (HLB) and are transmitted by *D. citri* are outside the orchard, because in this case large amounts of insecticides are used in the commercial orchards. *Tamarixia radiata*, the biological control agent, is being released in numbers of 3200 parasitoids/ha on about 12,000 ha each 15 days. *Tamarixia radiata* is released in areas at distances of up to 1.5 km outside the commercial orchards, in abandoned orchards (where HLB already occurred), organic areas, areas with *M. paniculata* (host-plant of the psyllid), and residential backyards.

For multiplication, that is, augmentative or applied biological control, it is necessary to produce millions of insects for release; these are the mass rearing, which involve the production of millions of biological control agents for field releases, with thousands of individuals per ha being released simultaneously. In this case, control is more rapid, more closely resembling the chemical products to which the farmer is accustomed.

Mass rearing began to be referenced in the book by Smith (1966) [14] and the compilation provided in a book by Singh (1977) [15] on artificial diets for insects. Other books on insect rearing techniques followed, such as those of Singh and Moore (1985) [16], Cohen (2003, 2015) [17,18], Schneider (2009) [19], Panizzi and Parra (2012) [20], and Morales-Ramos et al. (2014) [21]. These artificial diets facilitated the development of IPM because they allowed insects to be laboratory-reared for a wider range of studies, including studies of BC. These artificial diets were developed mainly for species of Lepidoptera, Coleoptera, and Diptera (although diets exist for other orders) and presently have the same formulations as the diets from 1970–1980. For example, Parra et al. (2021) [22] studied biological aspects and the spatial distribution of the *Spodoptera* complex at different temperatures, using the diet of Greene et al. (1976) [23]. Several definitions of mass rearing were compiled by Parra (2008) [24].

Finney and Fisher (1964) [25] defined mass rearing as the economical production of millions of beneficial insects in an assembly line, with the objective of producing the maximum number of fertile females, with minimum man-hours and space, in the shortest time possible, and at low cost. Mackauer (1972) [26] and Chambers (1977) [27] combined the economic with the biological aspect and defined mass rearing as the production of insects capable of reaching objectives such as an acceptable cost/benefit ratio and at least exceeding 10 thousand to 1 million times the mean productivity of native females. Leppla and Adams (1986) [28] described mass rearing as an automated activity in integrated installations, with the objective of producing a relatively large supply of good quality insects for distribution.

Therefore, millions of insects are being produced. Labor represents 70–80% of the cost of production. Problems with the quality of the insects produced, as well as the sanitary problems, increase with the size of rearing systems. Considering the extensive agricultural areas in Brazil (10,000, 50,000, or 100,000 ha planted with the same crop), automation is necessary to produce sufficient insects for release in these areas. Storage of the insects produced in mass rearing systems is essential, so that rearing is not interrupted when the insects are not being released in the field. After storage, they must be of equal quality to wild insects. Mass rearing is the result of upscaling from a smaller rearing system, the so-called research rearing used by researchers in universities and research institutes for basic studies, involving only one or two technicians to conduct it.

**Figure 1:**
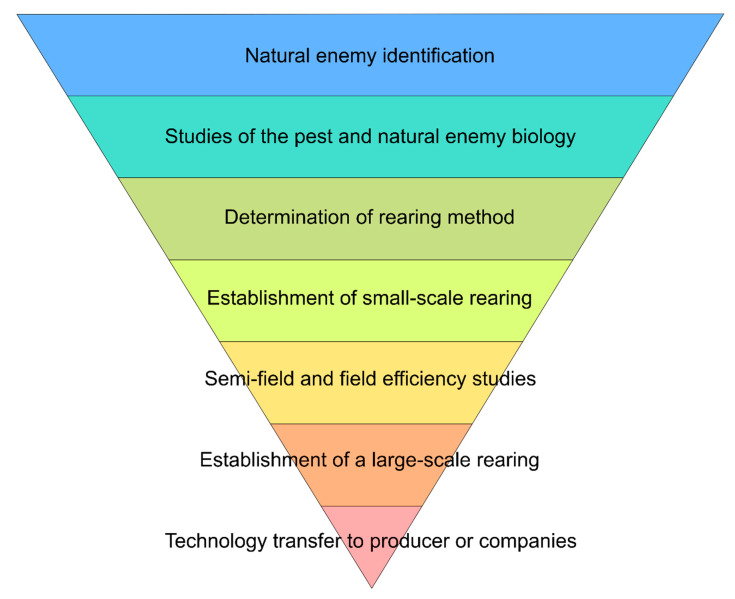
Stages of a biological control program, taken from Parra (2021) [31].

Considering the complexity and amplitude of studies necessary for a BC program, such a program must follow a certain logic [29,30]. It is suggested that a BC program follow a sequence of events, as cited by Parra (2021) [31] (Figure 1).

## 3. Forms/Means of Rearing

The production scheme can vary as a function of the cost or ease of rearing. The biological control agent can be reared in its natural host or a factitious host (term used for a host not natural, nevertheless, able to produce the parasitoid, i.e., alternative host). For example, in sugar cane, the exotic parasitoid *C. flavipes* is reared in larvae of *D. saccharalis*, its natural host. On the other hand, *T. galloi* is reared in a factitious host, eggs of *A. kuehniella*, instead of in eggs of the pest lepidopteran that it parasitizes (*D. saccharalis*), its natural host.

Analogously, the host and/or prey to be used in rearing the natural enemy can be reared on an artificial or natural diet (plants, flours, and stored grain). Although they are more laborious, biological control programs where an artificial diet is not viable can succeed well. In some cases, the plant that is actually targeted by the pest is used to rear the natural host, as in the case of *P. citrella*, reared on orange seedlings (the crop on which it is a pest) for use in rearing the parasitoid *A. citricola*. In other cases, non-target host plants are used, as in the case of *M. paniculata*, used to rear *D. citri* (both a pest and the vector of HLB), the natural host of the parasitoid *T. radiata*.

## 4. Successful Cases in Brazil with Different Forms of Rearing the Host and the Natural Enemy

### 4.1. Use of a Factitious Host to Rear a Parasitoid: Case Study of *Trichogramma* x Lepidopterans

At present, *Trichogramma* spp. are being used in more than three million ha. Successful use of these agents is closely linked to the ability to rear them in eggs of a factitious host [32]. By means of these factitious hosts, 50 to 400 thousand *Trichogramma* per hectare are being used in different crops in Brazil, at a cost comparable to agrochemicals. Thirty species of *Trichogramma* are known with natural occurrence in Brazil [33,34].

Research with *Trichogramma* in Brazil began in the 1940s, aiming to control the tomato fruit borer, *Neoleucinodes elegantalis* (Guenée) (Lep.: Crambidae), in tomato crops. This program was discontinued because the producers opted to use chemical insecticides instead [35]. After a hiatus in research, as well as in the use of *Trichogramma*, in the 1980s, a group from Minas Gerais, headed by Professor George Washington de Moraes of the Federal University of Minas Gerais, restarted studies with *Trichogramma* in Brazil, to control forest pests [36]. However, in both cases, the little information available in the literature mainly described the hosts and rearing techniques used.

One of the first successful, and best documented, cases was in the northeast region of Brazil, where *T. pretiosum* reared in *Sitotroga cerealella* (Olivier) (Lep.: Gelechiidae) was released on 1500 ha, with about 400,000 parasitoids released per hectare. This project was based on programs conducted by BC companies in Colombia. Research in Brazil followed, mainly at USP/ESALQ, inspired by the French model led by Dr. Jean Voegelé (INRA, France). As described in item two of this review, the study was carried out systematically and continuously, involving different areas of inter- and multidisciplinary research.

The principal stages of this project can be summarized as follows:

1. Collection, identification, and maintenance of *Trichogramma* strains;
2. Selection of a factitious host for mass-rearing the parasitoid;
3. Biological and behavioral studies of the parasitoid and the factitious host;
4. Population (eggs) dynamics of the target pest;
5. Mass rearing and quality control;
6. Release technique, number of parasitoids released, and release interval, as a function of plant physiology and number of release points;
7. Selectivity of agrochemicals;
8. Evaluation of efficiency;
9. Cost/benefit ratio.

The project, developed at USP/ESALQ, began by focusing primarily on the use of *Trichogramma* in sugarcane, a crop where BC was already used, with the braconid *C. flavipes* for controlling the cane borer *D. saccharalis*. The studies carried out in stages 1 and 2 allowed us to determine that the *Trichogramma* naturally occurring in eggs of *D. saccharalis* was a new species, named *T. galloi*, in tribute to Professor Domingos Gallo, precursor of studies on BC in Brazil and not *Trichogramma minutum* Riley (Hym.: Trichogrammatidae), as previously thought [37]. Further, life-table studies showed that the key factor in population growth of *D. saccharalis* is the egg stage [38]. After this selection of the species, the second step, i.e., selection of a factitious host for the rearing itself, was a watershed for the success of the program, as the eggs of *S. cerealella*, previously used in Brazil, are nutritionally poorer than eggs of *A. kuehniella* [39,40], as well as smaller. For this reason, acceptance by *T. galloi* is lower in this host. According to Gomes and Parra (1998) [41], *T. galloi* parasitizes 25% more *A. kuehniella* eggs than *S. cerealella* eggs.

After the most suitable host was determined, the next studies attempted to optimize the rearing of *A. kuehniella*. Early small-scale studies were based primarily on Strong et al. (1968) [42] and Bournier and Peyrelongue (1973) [43]. Thereafter, studies aimed at mass rearing were based mainly on the French model [44,45]. Parra et al. (1989) [46] found that a diet based on 97% whole-wheat flour and 3% brewer’s yeast was the most suitable for rearing *A. kuehniella*. However, a diet composed of 60% cornmeal (other than GMO) and 40% wheat flour proved to be similar in quality to the previously defined diet.

Stein and Parra (1987) [47] and Coelho Jr. (2011) [48] defined the thermal requirements, i.e., the thermal constant (K) expressed in degree-days and the lower thermal threshold of development or temperature threshold (Tt), of *A. kuehniella* as a thermal constant (K) of around 920 degree-days (DD), and a base temperature (Tt) of 9 °C. With this basic information, rearing became more predictable and manageable, making it possible to schedule the rearing cycle for production and release. Regarding the influence of temperature on rearing the factitious host, Coelho Jr. and Parra (2013a) [49] reported that *A. kuehniella* lays more eggs in more moderate temperatures, between 22 and 25 °C. Insects reared at higher temperatures (30 °C) during the immature phase, when transferred to 25 °C in the adult phase, their fertility was affected. Cerutti et al. (1992) [50] and Coelho Jr et al. (2016 a) [51] demonstrated that different densities of *A. kuehniella*/diet cause a temperature increase inside the rearing trays, which can reach up to 9 °C when the insect is between the 3rd and 4th instars. The authors demonstrated that both the density alone and the temperature increase are harmful to the laying capacity; therefore, egg densities, inoculated per kilogram of diet, must be between 0.2 g (7200 eggs) and 0.3 g (10,800 eggs). Cooling the rearing room to reduce the temperature increase on the 20th day also improves the insects’ laying ability [50,51]. Coelho Jr. and Parra (2013b) [52] demonstrated that trays of *A. kuehniella* can produce up to 23 mL of CO_2_ per hour; if accumulated in the breeding rooms, this CO_2_ can also be harmful, including to the technicians responsible for the rearing. Females of *A. kuehniella*, from rooms with a maximum CO_2_ concentration of 1200 ppm, laid 427 eggs, while those from rooms with a concentration of 4400 ppm produced 343 eggs.

**Figure 2:**
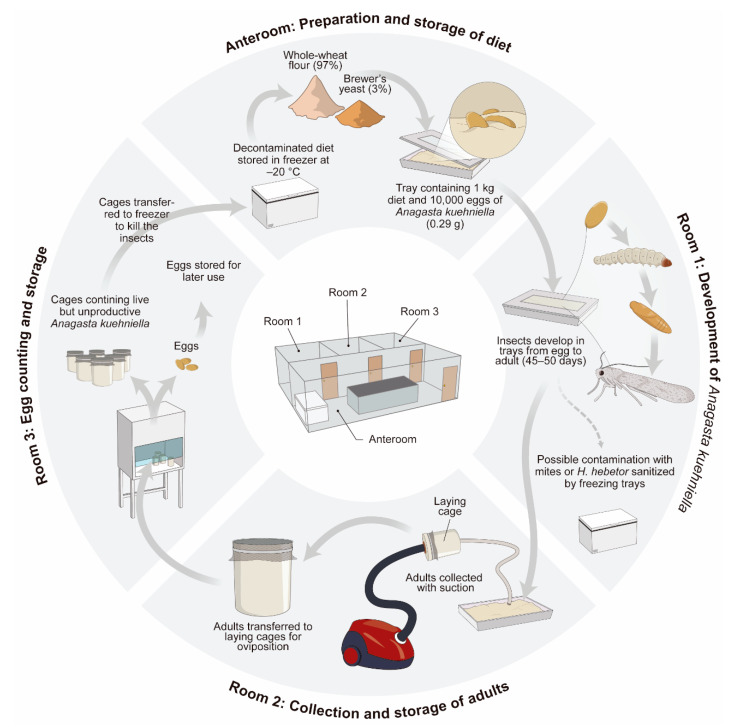
Rearing system for *Anagasta kuehniella* used in the Insect Biology Laboratory of USP/ESALQ; from Parra and Coelho Jr., 2021 [53].

The rearing system for *A. kuehniella*, used in the Insect Biology Laboratory of USP/ESALQ, is illustrated in Figure 2.

Currently, some companies in Brazil are capable of producing 10 to 20 kg of *A. kuehniella* eggs per day (1 g contains 36,000 eggs). The mastery of the technique for factitious host rearing is the basis for the success of these programs.

For rearing *Trichogramma* specifically, in addition to determining the most suitable host, studies sought to improve techniques for rearing it, especially on a mass scale, with a focus on controlling the quality of the insect. Morphological taxonomic confirmation was intensively investigated by Professor Roberto Antonio Zucchi, influenced by Dr. John Pinto (UCR, USA) and Dr. Bernard Pintureau (INRA, France) [54]. Molecular identification techniques were also studied [55], as well as identification by hyperspectral imaging [56].

*Trichogramma*, despite being a generalist insect, shows marked differences between strains of the same species, as is the case for *T. pretiosum*, which shows wide variation in its parasitism potential [57,58,59,60,61,62], flight propensity [63], and sex ratio [62]. Inbreeding depression and the founder effect were studied by Prezotti et al. (2004) [64], who showed that the insect is not subject to these biotic factors. To measure the flight capacity of mass-reared insects, an improvement in the flight-test device by Dutton and Bigler (1985) [65], was proposed by Prezotti et al. (2002) [66]. As mentioned above, it is important to rear the *Trichogramma* species and/or strains in isolation, without contamination between parasitoid species (due to their small size), which can lead to the failure of biological control programs (Danks, 1988) [67]. To prevent such contamination, cages with little air exchange are used, which can harm the rearing process. According to Coelho Jr. et al. (2017) [68], CO_2_ concentrations above 4.3% and O_2_ below 18.5% negatively affect some biological parameters, especially parasitism. Biological changes can occur in the development of insects stored at low temperatures, requiring studies to establish storage protocols, one of the principal problems in mass rearing. To better manage *Trichogramma* rearing, the thermal requirements of the different species were defined: *Trichogramma pretiosum* has a Tt of 12.8 °C and a K of 133.1 DD, *T. galloi* a Tt of 13.7 °C and a K of 131 DD, and *T. atopovirilia* a Tt of 13.5 °C and a K of 107.8 DD [69,70]. The appropriate relationship (proportion) between parasitoid and host eggs was also defined. To obtain the optimal parasitism, this number varies according to the species. In addition to the relationship (proportion), the time of exposure to parasitism must be taken into account. For *T. galloi* reared on *A. kuehniella*, the optimum ratio is three to four parasitoids to ten eggs of the alternate host for 24 h; for *T. pretiosum*, the ratio is 1:10 for the same period [71]. Egg density, therefore, leads to a concentration of kairomones that can affect parasitism, as observed by Lopes and Parra (1991) [72] and Pak and Oatman (1982) [73] under laboratory conditions, by Sá (1991) [74] in a greenhouse, and by Neil and Specht (1990) [75] in field conditions.

Today, more than 35 years after the program began, 11 companies are registered with the Brazilian Ministry of Agriculture, Livestock and Food Supply (MAPA) to sell the agents *T. pretiosum* and *T. galloi* in Brazil, in addition to the companies and cooperatives that maintain their own rearing operations. The most prominent crops treated are sugarcane, corn, soybeans, cotton, tomato, avocado, and melon, with numerous opportunities to expand the use of the parasitoid. The cost of applying 100,000 parasitoids per ha is 8–10 USD, adding another 2–3 USD for application via drone, for a total very close to the cost of agrochemicals.

It is very clear from the results presented that the basis of a BC program is a study of the insects’ biology, whether on a small or a large scale. Items 6 and 7 of the above list also depend on maintaining and rearing the insects in the laboratory. For quality control, constant maintenance of the insect laboratory population is essential, with periodic sampling to assess any changes in the natural enemy.

### 4.2. Use of a Non-Target Plant to Rear the Natural Host of Parasitoids: Example of *Diaphorina citri* x *Tamarixia radiata*

The biological control program for the insect vector of the disease, known as huanglongbing (HLB), is a peculiar type of biological control program, termed external management. The first case was cited by Parra et al. (2016) [76] and proven by Diniz et al. (2020) [13], in which the nymph parasitoid *T. radiata* was released outside citrus production areas to reduce the population of the vector pest *D. citri*. The number used is 3200 parasitoids/ha every two weeks during the citrus sprouting period [77]. The biological control program for *D. citri* required approximately 10 years to establish, from the beginning, with identification of the natural enemy in Brazil, until the determination of the cost/benefit ratio for using this strategy, following the steps in Figure 1 [76,78]. The natural enemy is reared in its natural host *D. citri*, which does not have an artificial diet, so *D. citri* is reared using plants. The plant used is the rutaceous *M. paniculata* [79], host of *D. citri*. *Murraya paniculata* is used because it is more financially viable; is more easily manipulated to produce shoots more continuously, which is necessary for the development of the pest (*D. citri*), and is a very favorable host for the pest in nature. *Murraya paniculata* has biological and financial advantages, compared with *Citrus* sp. L, as well as with *Murraya koenigii* (L.) (Sap.: Rutaceae) [80]. *Murraya paniculata* plants are kept in greenhouses with 50% shading, and sprouting is stimulated by pruning the apical third of the plant. After approximately one week, the plant with sprouts is suitable for infestation by the pest [81]. The rearing is carried out in cubical cages 47 cm on a side, at a ratio of six plants (with sprouts) to 300 psyllid adults. After seven days, the adult psyllids are removed from the cage, and the plants with eggs are kept in the same greenhouse for 12 days, the period in which the nymphs enter the optimal stage (4th to 5th instars) to be offered to the parasitoid [78].

**Figure 3:**
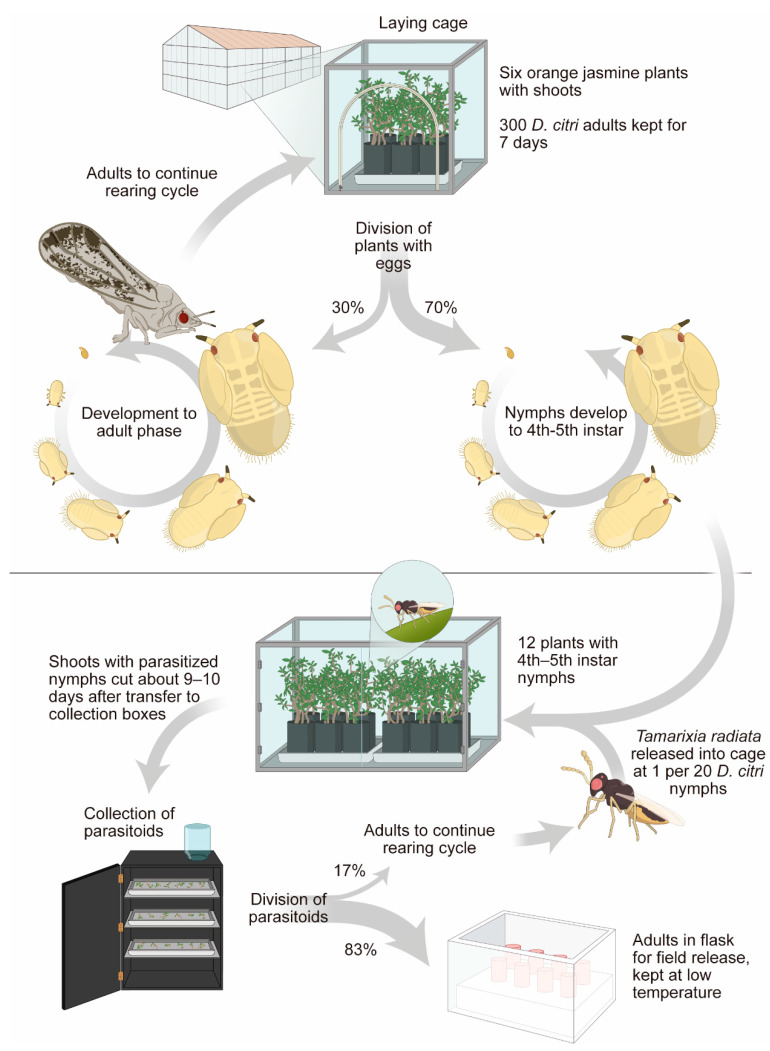
General production scheme for *Diaphorina citri* on *Murraya paniculata* plants, to rear its parasitoid *Tamarixia radiata*, according to the method developed at USP/ESALQ (Alves et al., 2014) [83].

The parasitism is carried out in a controlled condition of 25 °C temperature and 70% relative humidity. The ratio of one parasitoid to 10 nymphs is routinely used to optimize rearing. After 24 h of parasitism, *T. radiata* adults are removed from the cage; nine days after the parasitism, the branches containing the shoots with the pupae of *T. radiata* that developed in *D. citri* nymphs are cut off. The branches with the pupae are placed in a box, completely dark inside, with only one lighted opening in the top, where a 600-mL flask is placed to collect the adults of *T. radiata* [78]. These adults are kept in containers with food [honey:pollen in a 1:1 ratio] and transported to the release sites in Styrofoam boxes, with a temperature close to 18 °C. The release sites are in areas adjacent to commercial orchards or urban areas that use *M. paniculata* as an ornamental plant, among others (see item 2) [78] (Figure 3).

Currently in Brazil, 10 biofactories produce *T. radiata*, using the rearing model proposed by Parra et al. (2017) [82] and Diniz et al. (2021) [78]. Despite the lack of an artificial diet for the host and/or a factitious host, this system produces large numbers of parasitoids. The *T. radiata* BC program is part of an IPM program, recommended by the Citriculture Defense Fund (FUNDECITRUS).

### 4.3. Use of Plant (Natural Host) to Rear the Pest and Produce the Parasitoid: Example of *Phyllocnistis citrella* x *Ageniaspis citricola*

In Brazil, the citrus miner *P. citrella* was also a major phytosanitary problem in the mid-1990s. The pest was first recorded in the municipality of Iracemápolis, state of São Paulo, in 1996 [84]. This pest causes direct damage by feeding on the leaf mesophyll and, in addition, is an aggravating factor for the spread of citrus canker caused by the bacterium *Xanthomonas axonopodis* (Hasse) (Xan.: Xanthomonadaceae), which penetrates the leaves through the lesions caused by the insect.

In 1998, the natural enemy *A. citricola* was introduced in Brazil, brought from Florida, USA, in cooperation with Dr. Marjorie A. Hoy of the University of Florida. *Ageniaspis citricola* had been introduced into Florida from Australia, although it was originally described from Vietnam [85]. The rearing technique for the host *P. citrella* and parasitoid *A. citricola* was adapted from that performed at the University of Florida by Chagas et al. (2002) [86].

**Figure 4:**
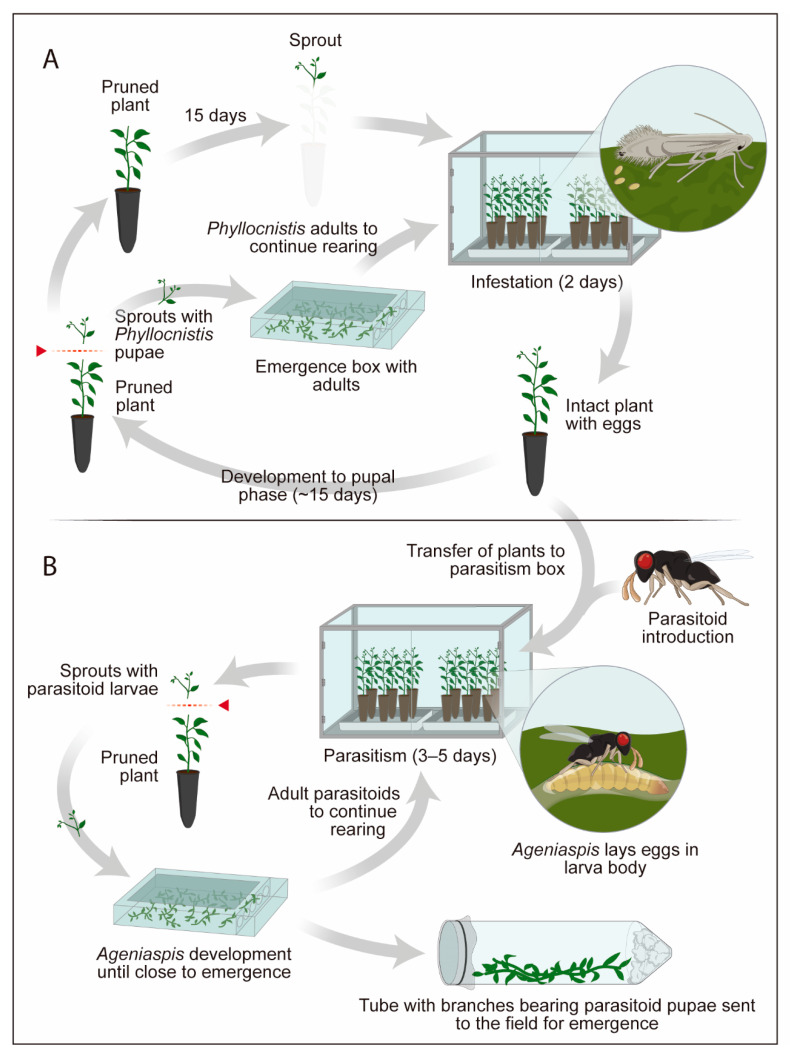
Production scheme for *Ageniaspis citricola* (**A**) natural host of *Ageniaspis citricola* (**B**), using plants of *Citrus limonia* (adapted from Chagas et al., 2002 [86]).

The citrus miner *P. citrella* was reared according to Chagas et al. (2002) [86] (Figure 4) in citrus plant seedlings. The species used most often was Rangpur lime, *Citrus limonia* Osbeck (Sap.: Rutaceae), approximately 25 to 30 cm tall, planted in plastic tubes 20 cm high by 1.5 cm in diameter. The top third of the plants was pruned, with fertilization to stimulate production of the shoots, which appeared after 15 days. The plants were then exposed to *P. citrella*, which oviposited preferentially on the shoots. The Rangpur lime seedlings in tubes were exposed to the miner infestation for 2 days, with one *P. citrella* couple for each group of three plants (1:3). The moths were fed with pure honey, placed on the glass walls of the cages. The pest’s egg-adult cycle under controlled conditions of 25 °C, and 70% RH is completed in 15 days. After the miner pupae were observed, they were collected by cutting the branches containing them, keeping the branches in plastic containers lined with moistened filter paper on the bottom, and covered with voile fabric to allow aeration. These containers are called “adult emergence boxes” [86].

The populations of *A. citricola* were kept in cages similar to those of *P. citrella* (Figure 4), using the same tube system for the production of pest eggs. The eggs were exposed to parasitism for a period of 4 to 5 days, the mean life-span of the parasitoid. The Rangpur lime plants containing the parasitized eggs were kept on 25 × 15 cm metal grids, immersed in plastic trays with water, at a constant temperature of 25 ± 2 °C, relative humidity of 70 ± 10%, and photophase of 14 h. Under these conditions, after 14 days, branches attacked by the miner containing pupae of *A. citricola* were cut. After 18 days, at 25 °C, adults of *A. citricola* were obtained (Figure 4).

The parasitoid was released in 1998, two years after the pest was found in the country, in citrus-producing municipalities in the state of Sao Paulo. Since *A. citricola* was successfully established in Brazil, the parasitoid has been released in other states, in different regions of the country. As of December 2003, approximately 850,000 parasitoids had been released in 67 citrus-producing municipalities in the state of São Paulo. The parasitoid has a high potential to disperse over 40 km in 45 days. The releases were carried out with 30 to 50 leaves bearing pupae of the parasitoid, considering that the insect is polyembryonic; therefore, this number of leaves covered an area of 25 ha. The parasitoid was recovered in the field, providing proof of its establishment, three months after the first releases [85]. In a survey conducted in 2004 (five years after the first releases), in an area of 18,500 ha, in 22 properties, in 17 municipalities, *A. citricola* was found in 100% of the areas surveyed [85].

### 4.4. Use of Natural Host Reared on Artificial Diet to Rear a Parasitoid: Example of *Cotesia flavipes* x *Diatraea saccharalis*

Currently, the largest biological control program being conducted in Brazil uses the larval parasitoid *C. flavipes* to control *D. saccharalis*. It is estimated that an area of 3.5 million hectares has been treated with this agent. The key factor in the successful use of *C. flavipes* is closely related to the ability to rear the natural host (*D. saccharalis*) on an artificial diet, based on casein and wheat germ. This diet was developed in the late 1960s by researchers from Louisiana State University, who aimed to study pest attractants [87]. In 1969, the diet was introduced into Brazil by Prof. Domingos Gallo (USP/ESALQ) for studies on the biological control of *D. saccharalis*, with the native tachinids *Lydella minense* (Townsend) (Dip.: Tachinidae) and *Billaea claripalpis* (Wulp) (Dip.: Tachinidae) [88]. In 1971, the larval parasitoid *C. flavipes* was introduced into Brazil, from Trinidad and Tobago for BC programs, and a strain from Pakistan was brought later [89].

The diet of Hensley and Hammond (1968) [87] was adapted to Brazilian conditions [90,91,92]. Parra and Mihsfeldt (1992) [93] demonstrated that a diet using white corn, adapted from the diet of Poitout and Bues (1970) [94], with a cost 2.7 times lower than the diet of Hensley and Hammond (1968) [87], which produces *D. saccharalis* with biological quality comparable to insects reared on the original diet. Since *C. flavipes* is a koinobiont parasitoid, a re-feeding diet was developed by adding an anti-contaminant (acetic acid) [95].

The rearing process for *C. flavipes* is still too labor-intensive and is conducted in the laboratories of sugar and alcohol production plants and private companies. One company, responsible for treating 10% of the total area covered, has 270 employees. The parasitism is still carried out manually, using mated females. The process of removing pupae cocoons from the re-feeding diet is also done manually, and one larva produces about 50–60 cocoons of *C. flavipes*. The search for automated procedures is continuing, but so far automation has not reached a satisfactory level. Quality control of the insects produced, both the host and natural enemy, is a routine part of the rearing process. One of the most serious problems is contamination of rearing colonies of the host *D. saccharalis* by the microsporidian *Nosema* sp. Nägeli (Dis.: Nosematidae) [96], which can decimate host populations and affect biological aspects of *C. flavipes* [97]. To prevent this problem, the anti-contamination agents, described in the literature, for the diet of *D. saccharalis* must be used (methyl parahydroxybenzoate (Nipagin) 0.14%; formaldehyde (37.2%solution) 0.04%), the sanitation conditions of the rearing colonies must be appropriate, and the initial colonies must be established from a microsporidian-free source.

After nearly 50 years of applied studies on rearing *C. flavipes* in *D. saccharalis* reared on an artificial diet, a total area of 3.5 million ha has been treated, with numbers of six to eight thousand parasitoids per ha. This treated area is comparable to some small countries.

### 4.5. Use of Natural Host Reared on Artificial Diet to Rear a Parasitoid: Example of *Telenomus podisi* x *Euchistus heros*

Currently in Brazil, expectations are high that the egg parasitoid *T. podisi* can be used to control *E. heros*, one of the key crop pests today. In the 1980s, egg parasitoids were used in soybean cultivation in small areas of organic soybeans and effected satisfactory control, reaching 98% parasitism [98]. In this program, stink bugs, especially *Nezara viridula* L. (Hem.: Pentatomidae), were controlled, mainly with the parasitoid *Trissolcus basalis* (Wollaston) (Hym.: Scelionidae) [99]. However, mass production of these natural enemies was hampered by the lack of an adequate diet. The stink bugs were reared on a natural diet and, after a few generations, the laboratory population degenerated [5].

Development of an artificial diet to meet the nutritional needs of stink bugs over generations has become the objective of a line of research aiming at BC of stink bugs. Research on an artificial diet for stink bugs started with Panizzi et al. (2000) [100], working with *N. viridula*. In this study, a semi-liquid diet, described by Kamano (1980) [101], for rearing the alydid *Riptortus clavatus* (Thunberg) (Hem.: Alydidae) in Japan, was adapted to Brazilian conditions. Later, Fortes et al. (2006) [102] and Siqueira (2007) [103] modified the diet, with the addition of oil and freeze-dried vegetables.

This objective was achieved after years of research, and the key milestone was the diet proposed by Mendoza et al. (2016) [5], who tested more than 13 diets and found that a diet based on freeze-dried beans and snap peanuts was nutritionally adequate and capable of producing *E. heros* of similar quality to the wild insect. *Euchistus heros* eggs are used to rear the parasitoid *T. podisi*, which does not have a factitious host. *Telenomus podisi* is reared by offering *E. heros* eggs, which can be stored in a freezer at −80 °C for up to one year, at a ratio of 20:1 (eggs:parasitoid) [104]. Four companies are currently registered to sell the parasitoid in Brazil, which has been released on 200,000 ha, still a very small area, compared to the total area of 40.2 million ha, cultivated with soybeans in Brazil [1]. The parasitoids are used on 6000 to 10,000 ha, beginning with the R1 stage of soybean. The application has been carried out with drones and/or manually, at a cost of approximately 10–12 USD per ha. To increase production of the hosts, there have been attempts to automate the rearing process, which is currently a bottleneck in the production of the parasitoid.

## 5. Final Considerations

The number of entomologists involved in insect rearing, especially mass rearing, in educational and research institutions is decreasing worldwide, with many specialists moving to other emerging areas, such as biotechnology, robotics, artificial intelligence, nanotechnology, etc. However, the successes in BC described here illustrate the need for a balance. Basic biological studies are needed on both biotic factors (mating, oviposition, adult feeding, and diapause) and abiotic factors (thermal and hygrometric requirements, photoperiod, and CO_2_). Continual maintenance of laboratory populations is necessary for studies of quality control, chemical selectivity, determination of the release time, number of release points, etc., so that mass rearing and successful biological control can be achieved.

Sometimes, the development of an artificial diet, such as the diet for *E. heros* to allow control with *T. podisi* [5], can be decisive in a pest control program. In any case, automation of rearing processes will undoubtedly be a huge leap forward in this program, considering that although the 200,000 ha that have been treated seems to be a large area, it is still very small, compared to the 40 million ha of soybeans in Brazil.

Rearing of insects, whether pests through artificial diets or on plants, or beneficial insects in factitious or natural hosts, in addition to being essential for BC programs, also has a preponderant role in IPM programs. As already mentioned, rearing of insects enables bioecological, biotechnological, behavioral, resistance-management studies, and selectivity and feasibility studies of entomopathogens. An example of the importance of rearing insects for IPM programs is the case of *Gymnandrosoma aurantianum* (Lima) (Lep.: Tortricidae), an important citrus pest in Brazil. The diet for this species was developed by Garcia and Parra (1999) [105]. After laboratory rearing methods were established, several studies were carried out, aiming toward the management of this pest; a study of the sexual behavior of the pest, reared on the diet, led to the synthesis of a synthetic sex pheromone, which guided the decision-making of citrus growers [106]. After 10 years of using the sex pheromone, citrus growers in the state of São Paulo avoided losses, estimated at up to 1.3 billion dollars in that period [107].

BC has been increasing in Brazil more than in the rest of the world, and the use of Agriculture 4.0 (precision and digital agriculture) has been decisive for this [108], including pests monitoring [109], the release of natural enemies and automation of mass rearing with modern technologies, such as reflectance [56], robotics, and artificial intelligence. In all cases, insect rearing and mass rearing continue to serve as the foundation for the success of biological control programs.

## Data Availability

Not applicable.
